# hADSCs derived extracellular vesicles inhibit NLRP3inflammasome activation and dry eye

**DOI:** 10.1038/s41598-020-71337-8

**Published:** 2020-09-03

**Authors:** Chaoqun Yu, Peng Chen, Jing Xu, Yaning Liu, Hui Li, Linna Wang, Guohu Di

**Affiliations:** 1 Department of Special Medicine, School of Basic Medicine, Qingdao University, 308 Ningxia Road, Qingdao, 266071 China; 2 Department of Anthropotomy and Histo-Embryology, School of Basic Medicine, Qingdao University, Qingdao, China; 3 Qingdao Haier Biotech Co.Ltd, Qingdao, China

**Keywords:** Mesenchymal stem cells, Conjunctival diseases, Corneal diseases

## Abstract

The present study was set out to address the therapeutic efficacy of human adipose tissue stem cells derived extracellular vesicles (hADSC-Evs) in a mouse model of dry eye disease and to investigate the underlying mechanisms involved. hADSC-Evs eye drops were topically administered to mice that subjected to desiccating stress (DS). Clinical parameters of ocular surface damage were assessed with fluorescein staining, tear production and PAS staining. For in vitro studies, cell viability assay and TUNEL staining were performed in human corneal epithelial cells (HCECs) treated with hADSC-Evs under hyperosmotic media. In addition, immunofluorescent staining, Real-time PCR (qRT-PCR) and Western blots were used to evaluated NLRP3, ASC, caspase-1, and IL-1β expression levels. Compared with vehicle control mice, topical hADSC-Evs treated mice showed decreased corneal epithelial defects, increased tear production, decreased goblet cell loss, as well as reduced inflammatory cytokines production. In vitro, hADSC-Evs could protect HCECs against hyperosmotic stress-induced cell apoptosis. Mechanistically, hADSC-Evs treatment suppressed the DS induced rises in NLRP3 inflammasome formation, caspase-1 activation and IL-1β maturation. In conclusion, hADSC-Evs eye drops effectively suppress NLRP3 inflammatory response and alleviate ocular surface damage in dry eye disease.

## Introduction

Dry eye disease (DED) is a highly prevalent ocular surface disorder in the world^1^. It is estimated that more than 16 million adults are diagnosed DED in US, and the prevalence in Asia is even higher than in western countries^2,3^. According to the reports of Dry Eye Workshop (DEWS II), DED is a multifactorial disease that characterized by loss of homeostasis of the tear film^4^. The tear film instability causes symptoms of discomfort, itching, eye irritation, glare and blurry vision, leading to a reduction in quality of life. Although the pathogenesis of DED is not yet fully understood, mounting evidence showed that the “vicious cycle of inflammation”, including tear film instability, tear hyperosmolarity, apoptosis of cornea/conjunctiva and elevated levels of pro-inflammatory cytokines, play a core driver in its initiation and progression^5,6^. Accordingly, most of the treatments to date are focused on reducing inflammation and restoring normal tear film^7^.

Mesenchymal stem cells (MSCs) are self-renewing multipotent stromal cells that can be isolated from mesenchymal tissues such as bone marrow, adipose, umbilical cord, as well as other tissues^8^. Due to their immunomodulatory and trophic characteristics, MSC-based therapeutic intervention has been explored in a variety of immune-mediated disorders, including dry eye disease^9–12^. Although most of the results were promising, safety issues regarding MSC-based therapy are still a matter of concern.

Extracellular vesicles, with nanosized diameter of 30–150 nm, are known as intercellular communication mediators by transferring various bioactive molecules (proteins, lipids and RNAs)^13^. Recent studies revealed that MSCs derived extracellular vesicles play an important role in biological functions of MSCs^14–16^. Indeed, MSC-derived Evs were shown as a promising cell-free alternative to MSC-based therapy for several ocular diseases, such as autoimmune uveoretinitis, corneal epithelial wound healing and glaucoma^17–19^. However, few studies yet reported the efficacy of MSC-derived EVs for the treatment of dry eye diseases.

Adipose-derived stem cells (ADSCs), a type of MSCs, can be isolated from stromal vascular fraction of adipose tissue. Compare to MSCs originating from other tissue, ADSCs are perceived as the most easily and abundantly acquired stem cells from adult tissue. Emerging evidence has confirmed that ADSCs can secrete high levels of extracellular vesicles and ADSC-Evs exhibit excellent immunomodulatory effects^20,21^. Thus, in the present study, we aimed to test the therapeutic effect of human adipose tissue stem cells derived extracellular vesicles (hADSC-Evs) on dry eye disease in vivo and in vitro and to determine the underlying mechanisms.

## Results

### Identification of hADSCs and hADSC-Evs

**Figure 1:**
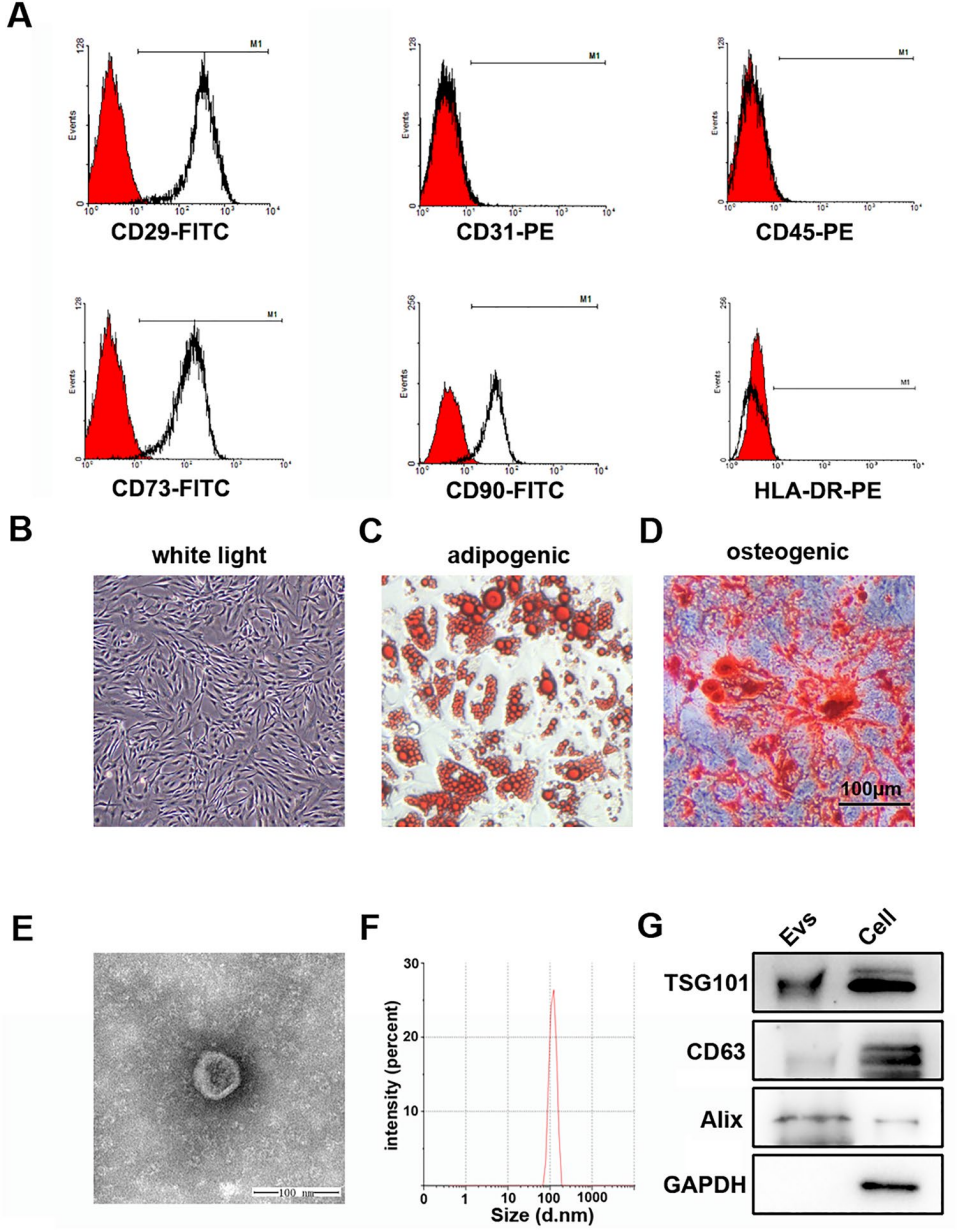
Identification of human adipose tissue derived stem cells (hADSCs) and extracellular vesicles derived from hADSCs (hADSC-EVs). (**A**) Flow cytometry analysis of surface markers in hADSCs. (**B**) Light microscope image of hADSCs at passage 2 (100 ×). (**C**,**D**) Adipogenic and osteogenic differentiation of hADSCs (100 ×). (**E**) Transmission electron micrograph of hADSC-EVs. (**F**) The size distribution of the hADSC-EVs was examined using Zetasizer Nano ZSP. (**G**) Detection of hADSC-Evs TSG101, CD63 and Alix expression by western blot.

hADSCs of passage 3 were tested for purity by flow cytometry for 3 independend times. As shown in Fig. 1A, they positively expressed CD29, CD73 and CD90, while negatively expressed CD31, CD45 and HLA-DR. In addition, hADSCs presented a homogenous population of spindle like cells (Fig. 1B). Botryoid lipid droplets were observed with Oil-Red-O staining after adipogenic induction (Fig. 1C) and positive staining of Von Kossa were shown after osteogenic induction (Fig. 1D). hADSC-Evs were collected and purified, which were cup-shaped with about 100 nm in diameter (Fig. 1E,F) and positive for exosomal markers TSG101, CD63 and Alix (Fig. 1G, Figs. S1–S4).

### hADSC-Evs can be taken up in vitro and in vivo

**Figure 2:**
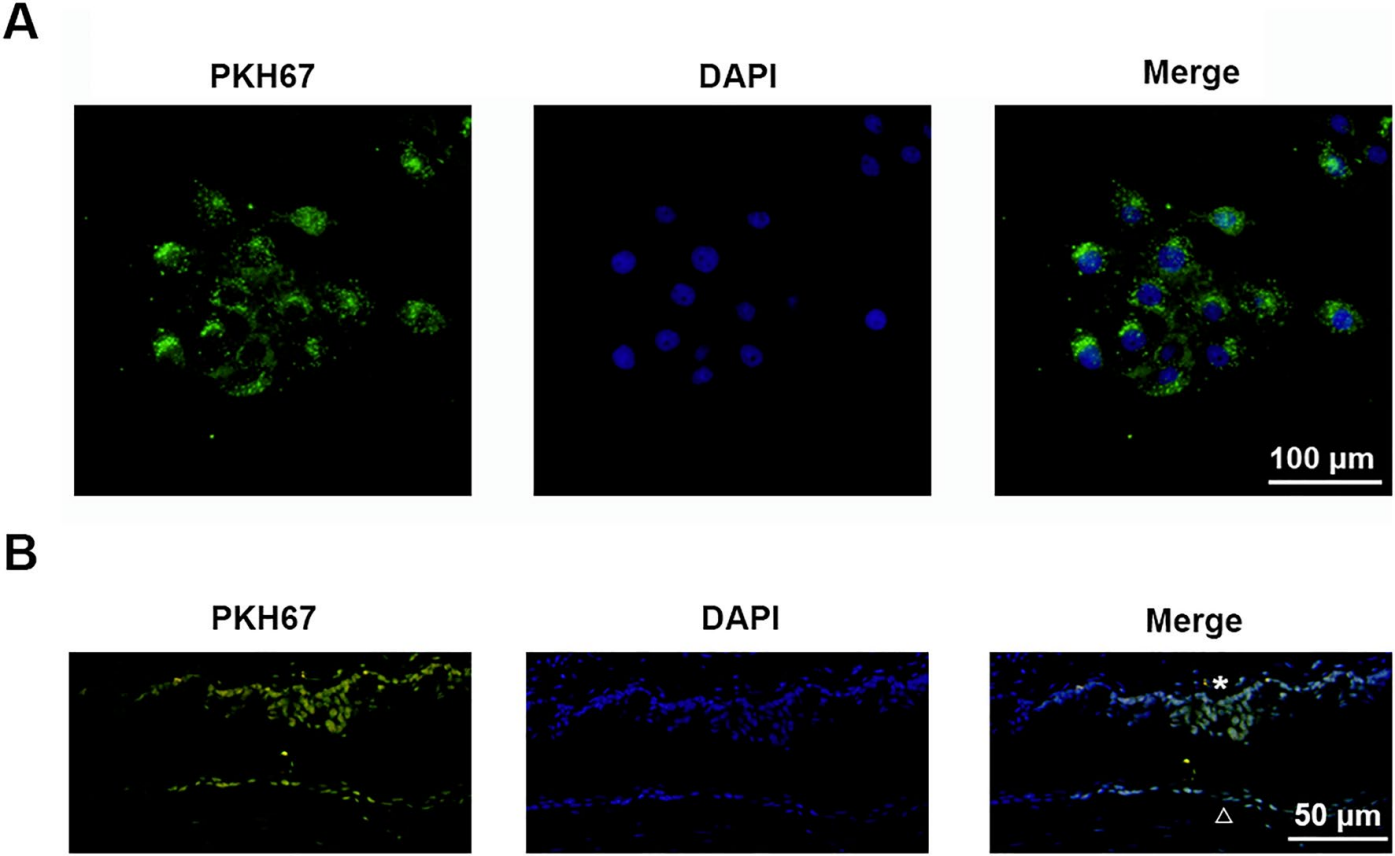
Uptake of hADSC-EVs in vitro and in vivo*.* (**A**) Fluorescent image of cultured HCECs incubated with PKH67 (green) labeled hADSC-EVs for 4 h. (**B**) Fluorescent image of mice cornea and conjunctiva following topical application of PKH67 labeled hADSC-EVs (*conjunctiva; open triangle cornea).

After labeled with PKH67 and incubated with HCECs for 4 h, hADSC-Evs with green fluorescence were observed in HCECs (Fig. 2A, Fig. S5), indicating the uptake of extracellular vesicles by HCECs. The uptake of hADSC-Evs was also evaluated in vivo following topical administration to murine ocular surface for 4 times. 2 h after the last treatment, eyeballs were snap frozen in Tissue-Tek optimum cutting temperature compound. Immunofluorescent staining of frozen corneal sections (7 μm thick) was performed and showed the distribution of labeled EVs in the corneal and conjunctival epithelium, indicating successful uptake of hADSC-Evs by corneal and conjunctival epithelium (Fig. 2B).

### Topical treatment of hADSC-Evs prevented desiccation-induced ocular surface damage

**Figure 3:**
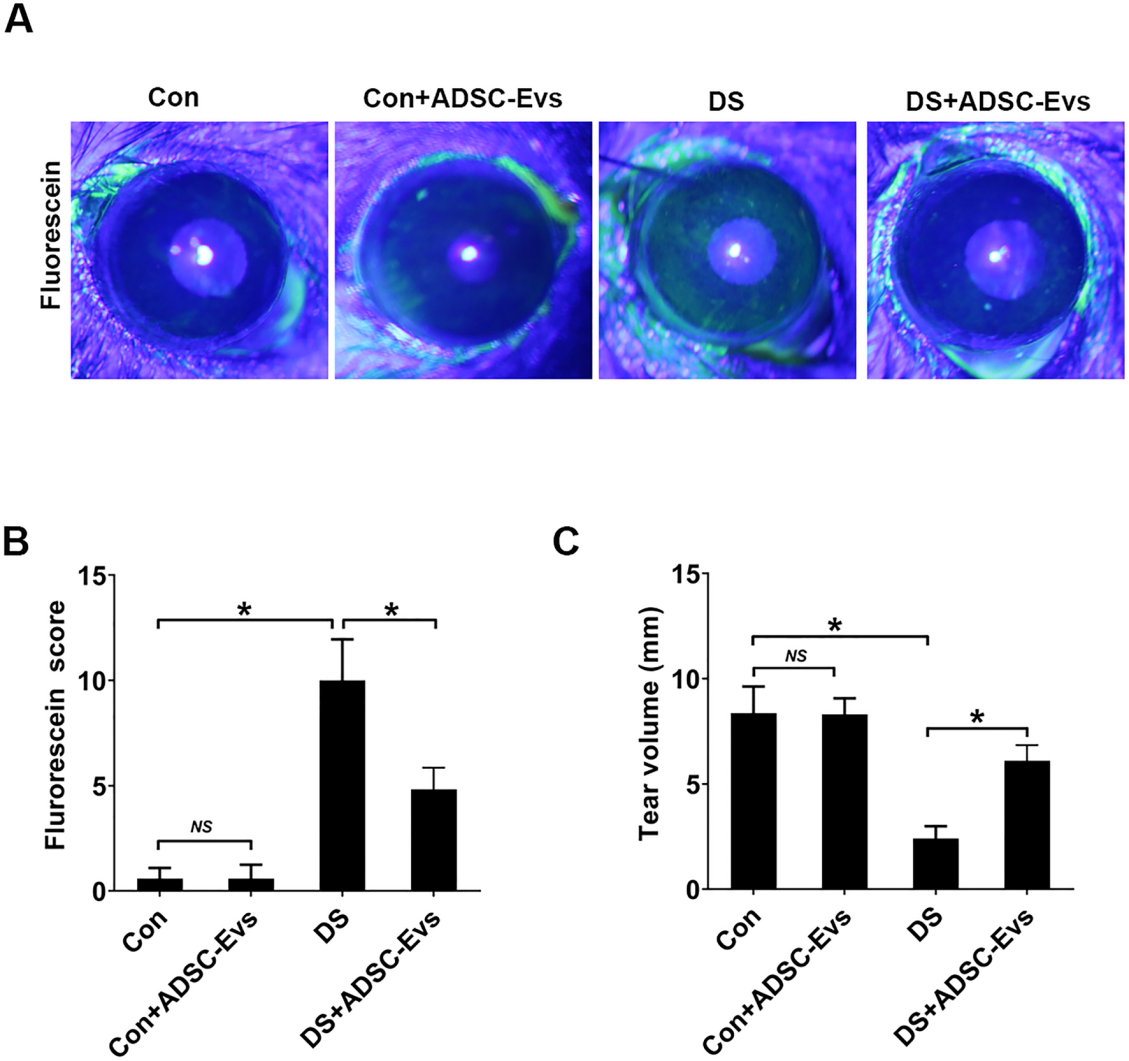
Effects of topical application of hADSC-EVs on DS-induced ocular surface damage. hADSC-Evs were topically applied four times daily under DS (DS + hADSC-Evs), while mice of DS group (DS) and normal control group (Con) received PBS. (**A**) Representative image of fluorescein sodium staining in cornea. (**B**) The mean score of fluorescein sodium staining. (**C**) Phenol red cotton test for the quantification of tear production. n = 6 mice per group, Data was shown as mean ± SEM. *p < 0.05.

**Figure 4:**
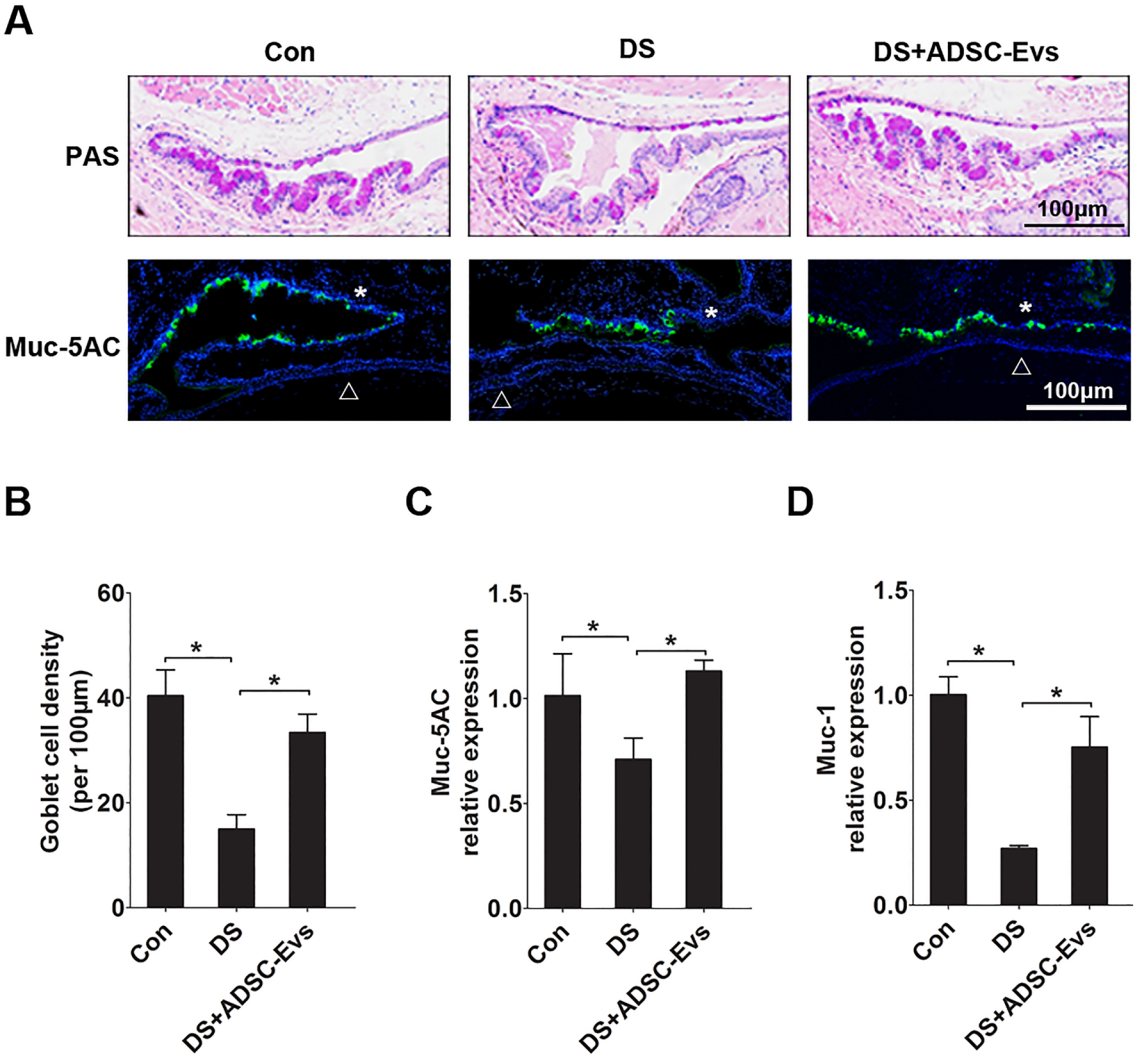
Effects of topical application of hADSC-EVs on DS-induced goblet cell dysfunction. (**A**) Representative image of PAS staining and Muc-5AC immunostaining in conjunctiva (*conjunctiva; open triangle cornea). (**B**) The density of goblet cells in the conjunctiva. (**C**,**D**) The mRNA levels of Muc-5AC and Muc-1 in conjunctiva (four conjunctivas were mixed as a sample). Data was shown as mean ± SEM. *p < 0.05.

After 5 consecutive days of desiccating stress exposure, corneal fluorescein score increased in vehicle (DS) group (12.0 ± 2.44 *VS* 1.2 ± 0.83), indicating the significant corneal epithelial defects. However, the hADSC-Evs group (5.2 ± 0.84 *VS* 12.0 ± 2.44) was markedly decreased 5 days after treatment (Fig. 3A,B). Moreover, normal mice treated with hADSC-Evs showed no difference, indicating the safety of hADSC-Evs. The phenol red cotton thread test was performed to evaluate the effect of hADSC-Evs eye drops on tear production. Compared to control group, the DS group showed markedly reduced tear production (2.40 ± 0.59 *VS* 8.36 ± 1.27 mm). While topical administration of hADSC-Evs significantly increased the tear production in mice under DS (6.10 ± 0.75 *VS* 2.40 ± 0.59 mm; Fig. 3C). Moreover, normal mice treated with hADSC-Evs showed no difference with vehicle group, indicating the safety of hADSC-Evs. We also observed significant decreases in the number of PAS-stained goblet cells in the conjunctiva after 5 days of DS (15.0 ± 2.74 *VS* 40.4 ± 4.93), whilst hADSC-Evs application significantly increased the number of conjunctival goblet cells under DS (33.4 ± 3.51 *VS* 15.0 ± 2.74; Fig. 4A,B). Furthermore, the topical eye drops of hADSC-Evs markedly upregulated the expression of Muc-5AC and Muc1 that suppressed under DS exposure (Fig. 4A,C,D).

### hADSC-Evs suppressed DS-induced apoptosis in vitro and in vivo

**Figure 5:**
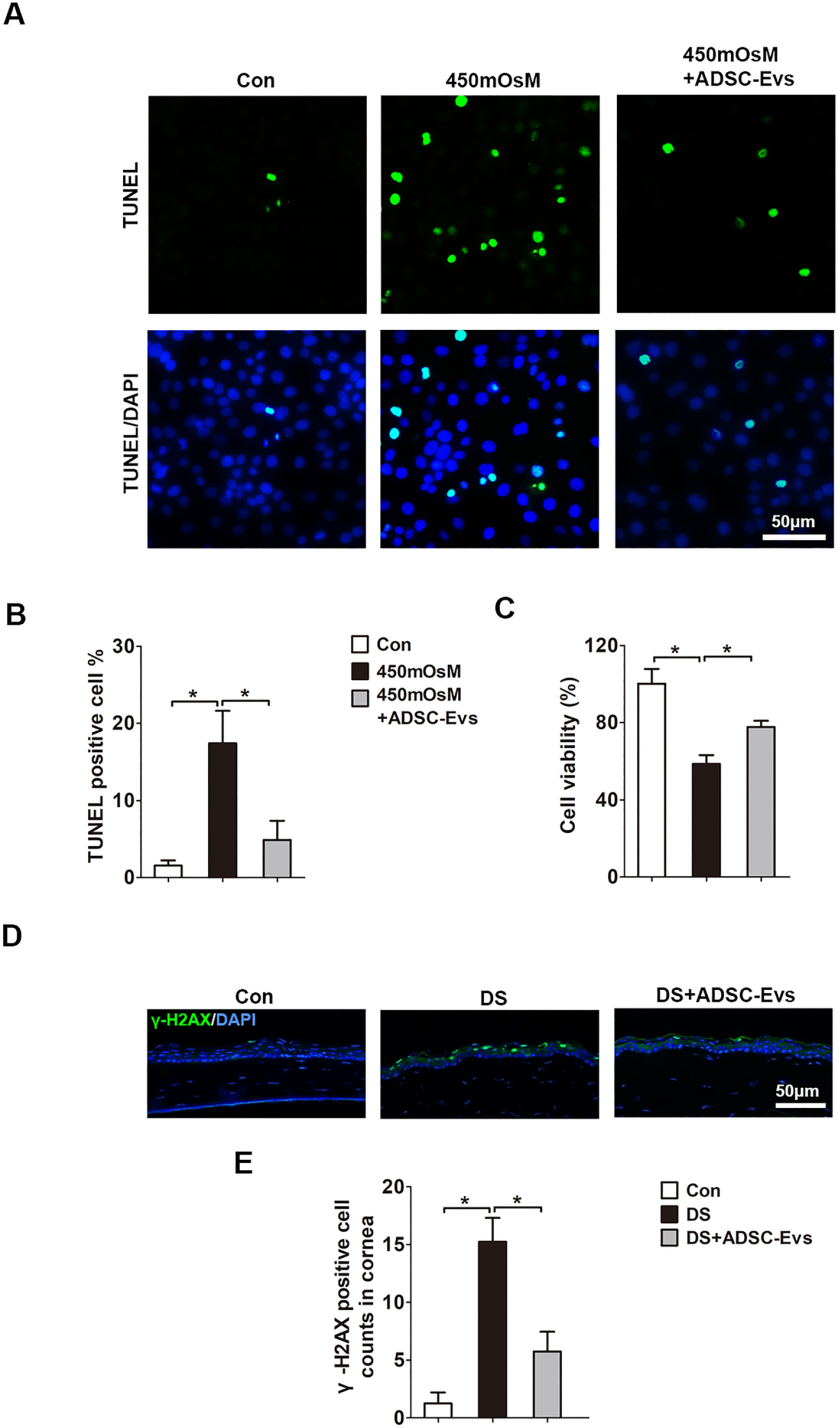
hADSC-Evs suppressed DS-induced apoptosis in vitro and in vivo*.* (**A**,**B)** Immunofluorescent staining of TUNEL positive cells under hyperosmotic stress (450 mOsM) or co-treated with hADSC-EVs. (**C**) Cell viability of HCECs treated with hyperosmotic medium or co-treated with hADSC-EVs. (**D**) Representative images of γ-H2AX staining in corneal epithelium. (**E**) The number of γ-H2AX-positive cells in corneal epithelium. Data was shown as mean ± SEM. *p < 0.05.

Previous studies have reported that DS causes cell oxidative damage and apoptosis^22,23^. As shown in Fig. 5A,B, the TUNEL positive cells were significantly increased in HCECs exposed to hyperosmotic medium for 24 h when compared to those under normal condition (17.42 ± 4.20% *VS* 1.55 ± 0.66%; *p* < 0.001). However, hADSC-Evs addition could inhibit cell apoptosis (4.88 ± 2.49% *VS* 17.42 ± 4.20%), which was further confirmed by cell viability assay (77.56 ± 3.80% *VS* 58.62 ± 3.79%; Fig. 5C). H2AX phosphorylation (γ-H2AX) is considered as a marker that indicated DNA damage and cell apoptosis. It has been shown that hyperosmotic stress induce γ-H2AX in corneal epithelial cells^24^. In consistent, as shown in Fig. 5D, the immunofluorescent staining demonstrates that the expression of γ-H2AX increased dramatically in corneal epithelium exposed to DS than that of normal control (15.20 ± 2.87 *VS* 1.25 ± 0.96). Whilst the positively stained cells were significantly decreased when treated with hADSC-Evs eye drops (5.75 ± 1.70 *VS* 15.20 ± 2.87; Fig. 5E).

### hADSC-Evs suppressed DS-induced NLRP3 inflammasome activation in vitro and in vivo

**Figure 6:**
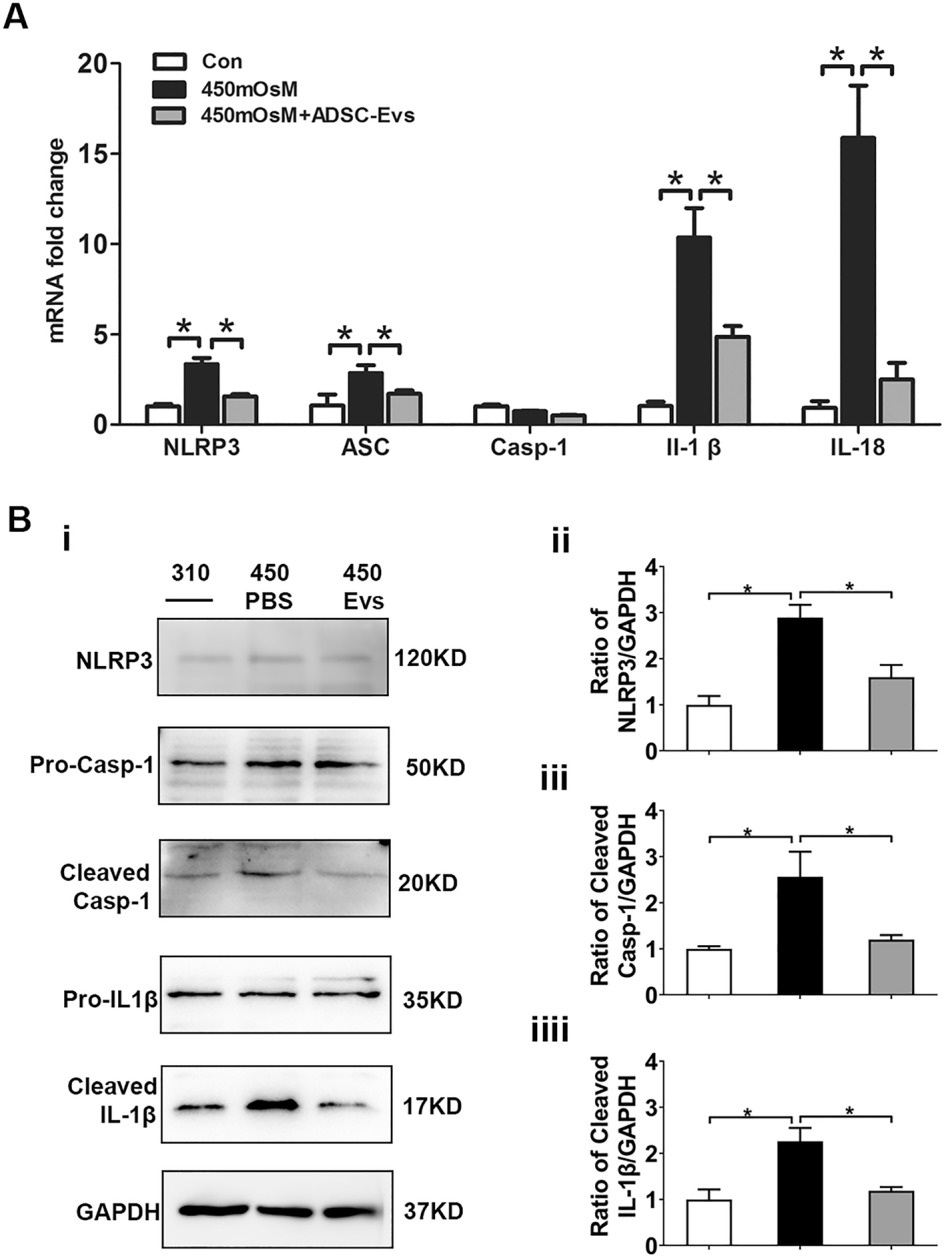
hADSC-Evs suppressed hyperosmotic stress-induced NLRP3 inflammasome activation in HCECs. (**A**) hADSC-EVs inhibited NLRP3 related gene expression in HCECs that under 450 mOsM hyperosmotic stress. (**B, i–iiii**) The protein levels of NLRP3 production, caspase-1 activation and IL-1β maturation were evaluated by Western blot, with GAPDH as internal control. Data was shown as mean ± SEM. *p < 0.05.

**Figure 7:**
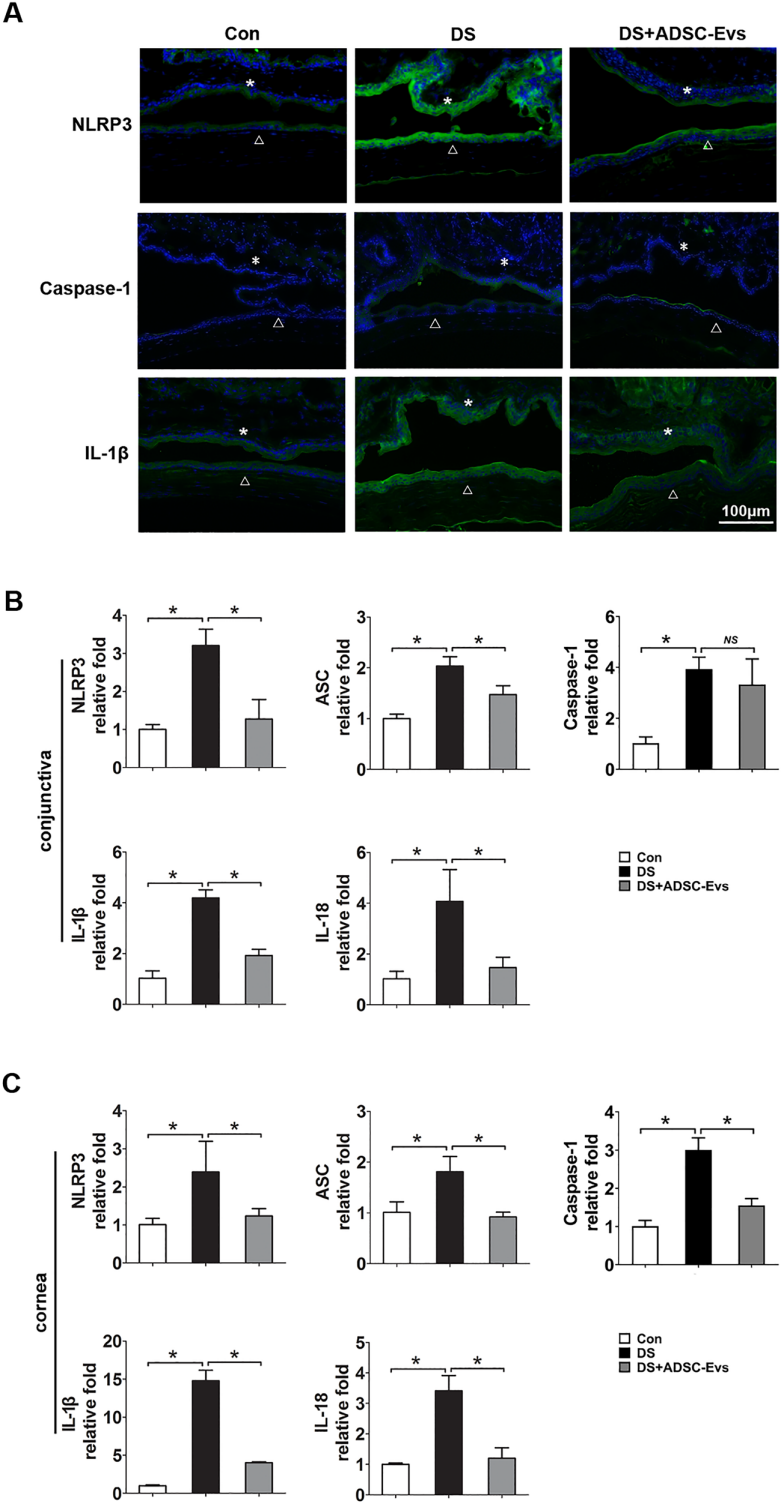
hADSC-Evs suppressed NLRP3 inflammasome activation in ocular surface of experimental mice under desiccating stress. (**A**) Representative image of immunofluorescent staining of NLRP3 related genes in corneal and conjunctival epithelia of desiccating mice or treated with hADSC-EVs (*conjunctiva; open triangle cornea). (**B**,**C**) qRT-PCR showed the mRNA levels of NLRP3 related genes in conjunctiva and cornea (four corneas/conjunctivas were mixed as a sample). Data was shown as mean ± SEM. *p < 0.05.

NOD-like receptors (NLRs), including NLRP3, are believed to participate in the pathogenesis of dry eye disease^25,26^. Our results showed that the mRNA expression levels of NLRP3, ASC, IL-1β and IL-18 were significantly raised under HS, while such increments were remarkably inhibited by hADSC-Evs (Fig. 6A). In agreement with the changes in gene expression levels, the NLRP3, cleaved caspase-1, and mature IL-1β protein expression levels also increased in the 450 mOsM group compared to normal control and declined in the hADSC-Evs group (Fig. 6B, Figs. S6–S10). Moreover, the immunofluorescent staining (Fig. 7A) and qRT-PCR (Fig. 7B,C) results clearly showed increased production ofNLRP3, caspase-1 and IL-1β in corneal and conjunctival epithelia of DS mice, whereas their levels decreased by hADSC-Evs application.

## Discussion

In the present study, our results revealed that topical administration of hADSC-Evs could be effective for the treatment of DED. Treatment of DED mice with hADSC-Evs showed decreased corneal epithelial defects, increased tear production, decreased goblet cell loss, as well as reduced inflammatory cytokines production. Moreover, hADSC-Evs could protect HCECs against hyperosmotic stress-induced cell apoptosis in vitro. To the best of our knowledge, this is the first study that evaluating the efficacy of topical application of hADSC-Evs in DED. Importantly, our data revealed that hADSC-Evs exert ocular surface protection through suppressing NLRP3 activation-mediated inflammation.

Recently, MSCs therapy is evaluated in clinical trials for the treatment of several ocular diseases and proved to be safe and efficacy^27,28^. The mechanism of MSCs therapeutic effect is mainly due to their paracrine activity. MSCs are reported as attractive source of EVs as they secrete a plethora range of therapeutic factors^29^. By delivering cytokines, enzymes, and microRNAs into recipient cells, MSC-EVs can regulate the pathological processes in several ocular disorders via different administrative routes. In experimental autoimmune uveoretinitis (EAU), systemic administration of MSC-EVs attenuated the onset of EAU via inhibiting activation of antigen-presenting cells and suppress the differentiation of T helper 1 (Th1) and Th17 cells^18^. In a genetic DBA/2J mice model of glaucoma, intravitreally delivery of BMSC-EVs provided significant neuroprotective effects through reducing the number of degenerating axons^17^. In mice corneal epithelial wound healing model, topical application of MSC-EVs promote corneal epithelial wound healing partially through enhancing epithelial cell proliferation and suppressing inflammation^19^. In consistent, our results revealed the protective effect of hADSC-Evs on DED treatment by topical administration. Of note, hADSC-Evs can be uptaken by corneal and conjunctival epithelial cells (Fig. 2A,B), suggesting hADSC-Evseye drops may be applied as an adjunctive therapy for ocular surface diseases.

Dry eye disease is clinically characterized by tear film instability. Dysfunction of the lacrimal function unit (LFU) alters the tear film composition, breaks ocular surface homeostasis and facilitates ocular surface inflammation^4^. Goblet cells and mucins are important components of LFU^30^. In line with other reports, our study revealed that chronic desiccating stress cause loss of goblet cell density and reduced expression of MUC-1 and MUC-5AC, while significantly improved by topical application of hADSC-Evs eye drops, indicating that hADSC-Evs protect ocular surface against desiccating stress via restoring normal tear film.

Recent studies have suggested that inflammatory response play a key role in the pathogenesis of dry eye disease^6,31^. Nucleotide-binding oligomerization domain (NOD)-like receptors (NLRs) are cytosolic sensors that can recognize extracellular or endogenous danger signals. Among them, NLRP3 inflammasome is well studied, which regulates the maturation of IL-1β^32^. Accumulating evidence suggest that NLRP-3-IL-1β signaling pathway play a priming role in DED progression^25,26,33^. Also, in ocular surface samples of DE patients, NLRP3 gene expression and IL-1β secretion were upregulated^34^. Here we demonstrated that hADSC-Evs application block the upregulation of NLRP3 and increased maturation of IL-1β HCECs in response to hyperosmotic stress, which was confirmed by corneal and conjunctival samples of DED mice, indicating that hADSC-Evs alleviated DED progression via inhibiting NLRP-3-IL-1β signaling axis. Indeed, previous studies have reported that MSC-EVs therapy for acute liver failure (ALF) through reduction of NLRP3 inflammasome^35,36^. However, the precise mechanisms of how hADSC-Evs regulate NLRP3 activation were not detected in this study, which deserves future investigations.

In conclusion, our findings showed that topical instillation of hADSC-Evs could protect the ocular surface by suppressing NLRP3 inflammasome activation in DED, and suggest that hADSC-Evs may represent a novel therapeutic approach in the management of DED and other inflammatory ocular disorders.

## Methods

### Cell culture and treatments

The hADSCs were isolated and cultured as our previous description^37^. The suspension of single cells was cultured in commercial kit (Cat#HUXMD-90011, Cyagen, Guangzhou, China) in a humidified atmosphere of 5% CO2 at 37 °C. Cells of passage 3–5 were used for all the experiments. The phenotype of ADSCs was determined by flow cytometer (BD Accuri C6 Plus, BD Biosciences, Franklin Lakes, USA) using specific antibodies as follows: CD29-FITC (Cat# 11-0299-41;ThermoFisher Scientific, MA, USA), CD31-PE (Cat#303105; Biolegend, San Diego, USA), CD45-PE(Cat#368509; Biolegend), CD73-FITC (Cat#344015; Biolegend), CD90-FITC (Cat#328107; Biolegend), HLA-DR-PE (Cat#307605; Biolegend). The differentiation potential of ADSCs was investigated by Human Mesenchymal Stem Cell Functional Identification Kit (Cyagen) in accordance with the manufacturer’s instructions. The stained cells were visualized using an Eclipse TE2000-U microscope (Nikon, Tokyo, Japan).

The immortalized human corneal epithelial cell line (HCEC) was cultured as previously described^38^. For hyperosmotic exposure, Confluent cultures were switched to serum free medium for 24 h, and treated for 6–12 h in medium with iso- and hyper-osmotic medium (450 mOsM) with adding 0 and 70 mM sodium chloride (NaCl). The osmolarity was measured by an osmotic pressure gauge (Tianhe analytical instrument Co., Ltd. Tianjin, China). The Cell cultured in 450 mOsM medium were simultaneously treated with or without hADSC-Evs (10 μg/mL). The cells treated 6 h were lysed in Lysis buffer from NucleoSpin RNA kits (BD Biosciences) for total RNA extraction. The cells treated for 12 h were lysed in RIPA buffer for western blot.

### Isolation of hADSC-Evs

Isolation of hADSC-Evs were performed as previously described with modification^21,39^. Briefly, when hADSCs reached 70–80% confluency, the culture medium was replaced with serum free medium and cultured for 48 h. The supernatants were centrifuged at 3,000×*g* for 30 min to discard cell debris and large vesicles, and filtrated through 0.22 µm filters. The supernatant (20 mL) was added to an Amicon Ultra-15 Centrifugal Filter Unit (100 kDa; Millipore, Billerica, USA) and centrifuged at 4,000×*g* to about 2 mL. hADSC-Evs were isolated from the supernatants using Exoquick-TC™ (System Biosciences, CA, USA) according to manufactory’s instruction.

### Characterization of hADSC-Evs

To evaluate the morphology, hADSC-Evs were suspended in 2.5% glutaraldehyde (1 mg/mL at protein concentration), dropped in carbon-coated copper grids, stained with 2% uranylacetate, dried, and examined by transmission electron microscopy (JEM 2100F TEM, Japan). To evaluate the size distribution, diluted hADSC-Evs (1 mg/mL at protein concentration) were subjected to Zetasizer Nano ZSP (Malvern Panalytical, Malvern, UK) and analyzed by the software equipped within the instrument. For western bloting, we detected extracellular vesicles biomarkers including CD63(1:1,000; Cat#ab134045, Abcam, Cambridge, UK), TSG101(1:1,000; Cat#ab125011, Abcam), Alix (1:1,000; Cat#ab186429, Abcam), and GAPDH (1:3,000; Cat#KC-5G5, Aksomics, Shang’hai, China).

### Extracellular vesicles labeling and tracking in vitro and in vivo

To detect the direct transfer of extracellular vesicles into HCECs, hADSC-Evs were labeled using PKH67 Fluorescent Cell Linker Kit (PKH67) (Cat#MINI67-1KT, Sigma, Saint Louis, USA), and then incubated with HCECs for 4 h, followed by fixation and (imaging. To evaluate the uptake of extracellular vesicles in vivo, normal female mice were topically applied with PKH67-labled hADSC-Evs (1 μg/μL) with 4 times a day (8:00 am; 11:00 am; 14:00 pm; 17:00 pm; n = 4; 5 μL/eye/time). 2 h after the last treatment, eyeballs were fully rinsed by PBS and snap frozen in Tissue-Tekoptimum cutting temperature compound. Immunofluorescent staining of frozen corneal section (7 μm thick) was performed and observed under microscope (Nikon).

### Mouse model of dry eye

This research protocol was approved by the Experimental Animal Ethics Committee of Qingdao University, and conformed to the standards of the ARVO Statement for the Use of Animals in Ophthalmic and Vision Research. Female C57BL/6 mice with 8–10 weeks age were purchased from Ji’nan Pengyue Experimental Animal-Breeding Co., Ltd. (Ji’nan, Shandong, China). Desiccating stress (DS) was created as previously described^23^. Briefly, mice were injected subcutaneously with 0.5 mg/0.2 mL scopolamine hydrobromide (Meilubio, Dalian, China) four times a day and exposure to an air draft and ≤ 30% ambient humidity for 5 consecutive days. Control mice were age- and gender-matched, and maintained in a normal environment at 50%-75% relative humidity (n = 6 per group). To evaluate the effect of hADSC-Evs on ocular surface damage of dry eye, 5μL hADSC-Evs (1 μg/μL) were topically applied four times daily with or without under DS (DS + hADSC-Evs) for 5 consecutive days, while mice of DS group and normal control group (Con) received vehicle (PBS).

### Corneal fluorescein staining

Corneal fluorescein staining was used to evaluate the barrier function damage of corneal epithelium. Briefly, 0.1 mL 0.25% fluorescein sodium (Jingming, Tianjin, China) were applied topically to the cornea. The eyes were blinked several times, rinsed with normal saline, and photographed using slit-lamp microscopy (66 Vision-Tech Co., Ltd., Suzhou, China) in cobalt blue light. For grading of the fluorescein staining, the cornea was divided into four quadrants, which were scored respectively. The 4 scores were summed to arrive at a final grade for each eye (minimum = 0, and maximum = 16). The fluorescein score was analyzed as previously described^22^ and as follows: absent, 0; slightly punctate staining less than 30 spots, 1; dense punctate staining more than 30 spots, 2; severe diffuse staining, but no positive plaque, 3; positive fluorescein plaque, 4.

### Measurement of tear production

Tear production (12 eyes of six mice per group) was determined with phenol red-impregnated cotton threads (AYUMI Pharmaceutical Corporation, Tokyo, Japan) as previous description with minimal modification^40^. Briefly, the thread was placed in the medial of the lower conjunctival fornix for 20 s. The length of the wet red thread was photographed and measured.

### PAS staining

The eyeballs were collected, fixed with 4% paraformaldehyde (PFA), paraffin embedded, sectioned at a 5 μm thickness, and stained with PAS staining kit (Cat#1008, Servicebio, Wuhan, China) according to the manufactory’s instruction. The superior and inferior conjunctiva were examined and photographed with a digital light microscope (Nikon). Three sections from the central parts of the eye from each animal were studied. Six mice were used for each group.

### Immunofluorescent staining

Eyeballs were snap frozen in Tissue-Tek optimum cutting temperature compound (Sakura Finetek, Tokyo, Japan). Immunofluorescent staining of frozen corneal section (7 μm thick) was performed using our previous methods^41^. Primary rabbit polyclonal antibodies against human and mouse NLRP3 (1:100; Cat#NBP1-77080, Novus Biologicals, Littleton, USA), caspase-1 (1:100; Cat#ab179515, Abcam), Mucin-5AC (1:100; Cat#SC-16903, Santa Cruz Biotechnology, Dallas, TX), γH2AX (1:100; Cat#ab2893, Abcam) and IL-1β (1:100; Cat#ab9722, Abcam) were used. Alexa-Fluor488 conjugated secondary antibodies (1:200; Cat#A-11037, ThermoFisher) were applied. Rabbit or goat IgG isotype control antibodys (1:200, Cat#ab172730, Abcam; 1:200, Cat#31425, Thermofisher) was used to excluded nonspecific staining and 4′ 6-diamidino-2-phenylindole (DAPI; Cat#H-1200; Vector, Burlingame, CA, USA) was used for nuclear counterstaining. For TUNEL staining, HCEC cultures on 8-chamber slides were performed by In Situ Cell Death Detection Kit (Cat#12156792910, Roche Diagnostics GmBH, Mannheim, Germany) according to the manufactory’s instruction. All staining was observed under microscope (Nikon).

### Real-time PCR

Total RNA was extracted from cultured cells, or from corneal and conjunctival tissue (four corneas/conjunctivasmixed as one sample) using the Nucleospin RNA Kits (Thermofisher). 1 μg of isolated RNA was reverse transcribed into cDNA using the Primescript First-Strand cDNA Synthesis kit (TaKaRa, Dalian, China). Quantitative PCR was performed using SYBRGreen reagents (Roche) as described in our previous reports^41,42^, with non-template controls were used. The primers information was listed in the supporting information (Table S1). The raw data was analyzed with the Bio-Rad CFX Maestro1.1 (Bio-Rad Laboratories, Philadelphia, USA). Ct values for each sample were obtained and further analyzed by GraphPad Prism software (version 5.0). GAPDH was used as an internal control. All the experiments were repeated independently for at least 3 times.

### Western blotting

Total protein was extracted from the lysed samples of HCEC cells in RIPA buffer containing protease inhibitors (Beyotime, Jiangsu, China). Western blotting was performed as our previously description^42–44^, with the following antibodies including: NLRP3 (1:1,000; Cat#ab263899, Abcam), caspase-1 (1:1,000; Cat#A0964 Abclonal, Wuhan, China), IL-1β (1:1,000; Cat#A11369, Abclonal) and anti-rabbit IgG HRP-linked Antibody (1:3,000; Cat#7074, Cell Signaling Technology, MA, USA). The specific bands were visualized by an enhanced chemiluminescence reagent kit (Cat#34094, Thermofisher). The western blot signals are 16-bit images captured by a Luminescent Imaging Workstation (Tanon, Shanghai, China).

### Statistical analysis

All the data in this study were representative of at least three independent experiments and presented as mean ± SEM. Statistical analysis was performed by GraphPad Prism software (version 5.0 GraphPad Software, Inc., San Diego, USA) using Student’s t-test for two group comparison or1-way ANOVA for groups more than three. Differences were considered statistically significant at *p* < 0.05.

## Supplementary information

Supplementary Information

## Data Availability

All data generated or analysed during this study are included in this published article (and its Supplementary Information files).
