# Supplementary material for: hADSCs derived extracellular vesicles inhibit NLRP3inflammasome activation and dry eye
Source: Sci Rep. 2020 Sep 3;10:14521. doi: 10.1038/s41598-020-71337-8 (PMC7471690; doi:10.1038/s41598-020-71337-8)
Supplement: Supplementary file 1 — Supplementary Information [file 41598_2020_71337_MOESM1_ESM.pdf]

hADSCs derived extracellular vesicles inhibit NLRP3inflammasome activation and dry eye

Chaoqun Yu[1#], Peng Chen[2#], Jing Xu[1], Yaning Liu[2], Hui Li[3], Linna Wang[3], Guohu Di[1]*

1. Department of Special Medicine, School of Basic Medicine, Qingdao University, Qingdao, China.

2. Department of Anthropotomy and Histo-Embryology, School of Basic Medicine, Qingdao University, Qingdao, China.

3. Qingdao Haier Biotech Co.Ltd, Qingdao, China.

# These authors contributed equally

*Correspondence author: Guohu Di, Ph.D

Qingdao University, 308 Ningxia Road, Qingdao, 266071, China.

Email: guohu_di@163.com,

Tel: 86-532-83780012 Fax: 86-532-83780010

Supporting information:

Table S1. Gene-specific primers used in the qPCR.

Gene	Accession number	Primer sequences
h-GAPDH	NM_002046.7	Forward:CATGTTCGTCATGGGTGTGAA
		Reverse:GGCATGGACTGTGGTCATGAG
h-NLRP3	NM_004895.4	Forward:TGGGTTTACTGGAGTACCTTTCG
		Reverse:GTAGCGTTTGTTGAGGCTCACA
h-ASC	NM_013258.5	Forward:CTTATCGCGAGGGTCACAAAC
		Reverse:CCTTGCAGGTCCAGTTCCA
h-CASPASE-1	NM_033292.3	Forward:CATCCCACAATGGGCTCTGT
		Reverse:TCTTTCAGTGGTGGGCATCTG
h-IL-1B	NM_000576.2	Forward:CTGAGCACCTTCTTTCCCTTCA
		Reverse:TGGACCAGACATCACCAAGCT
h-IL-18	NM_001562.3	Forward:CACCCCGGACCATATTTATTATAAGT
		Reverse:TGTTATCAGGAGGATTCATTTCCTT
m-GAPDH	NM_001289726.1	Forward:GCCACCCAGAAGACTGTGGAT
		Reverse:GGAAGGCCATGCCAGTGA
m-NLRP3	NM_145827.4	Forward:CTGCGGACTGTCCCATCAAT
		Reverse:AGGTTGCAGAGCAGGTGCTT
m-Asc	NM_023258.4	Forward:TGGACGCCATAGATCTCACTGA
		Reverse:CTGCCACAGCTCCAGACTCTT
m-CASPASE-1	NM_009807.2	Forward:CTGGGACCCTCAAGTTTTGC
		Reverse:CCCTCGGAGAAAGATGTTGAAA
m-IL-1B	NM_008361.4	Forward:CTTTCCCGTGGACCTTCCA
		Reverse:CTCGGAGCCTGTAGTGCAGTT
m-IL-18	NM_008360.2	Forward:GACCAAGTTCTCTTCGTTGACAAAA
		Reverse:CTATCCTTCACAGAGAGGGTCACA
m-MUC-5AC	NM_010844.3	Forward:GCTGCCCGACCCAGAACT
		Reverse:AGCTGACCATAAAGACATCGGAAA
m-MUC-1	NM_013605.2	Forward:GTGCCGCCGAAAGAGCTAT
		Reverse:CCTGCCGAAACCTCCTCATA

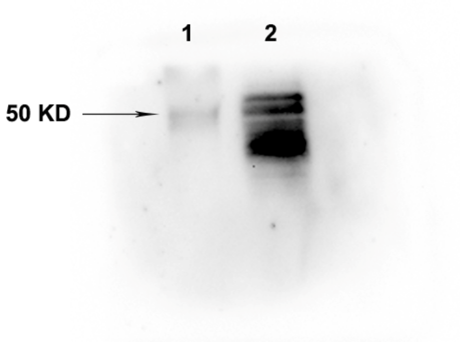

Figure S1: Full length gel of western blot for CD63. Lane 1: hADSC-Evs; Lane2: hADSC cell lysate.

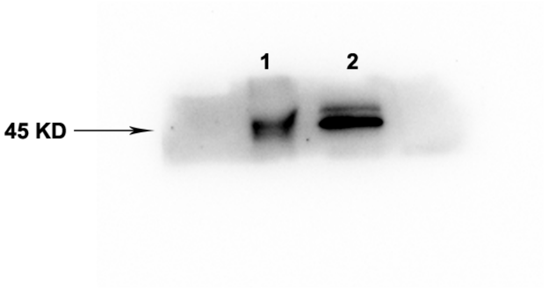

Figure S2: Full length gel of western blot for TSG101. Lane 1: hADSC-Evs; Lane2: hADSC cell lysate.

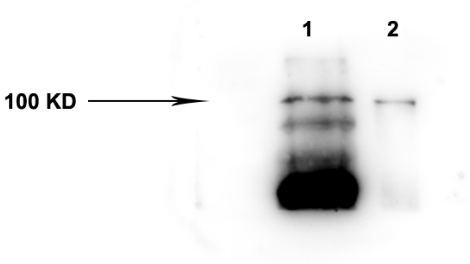

Figure S3: Full length gel of western blot for Alix. Lane 1: hADSC-Evs; Lane2: hADSC cell lysate.

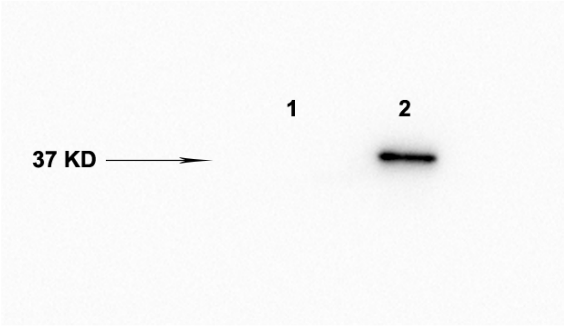

Figure S4: Full length gel of western blot for GAPDH. Lane 1: hADSC-Evs; Lane2: hADSC cell lysate.

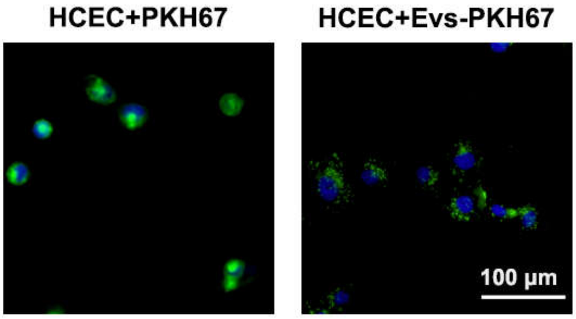

Figure S5: representative images of HCEC cells stained with PKH67 only *vs* cells uptaken Evs-PKH67(green:PKH67; blue:DAPI).

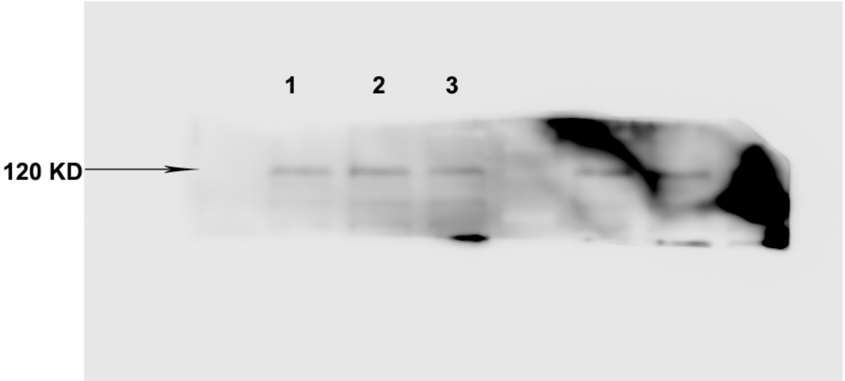

Figure S6: Full length gel of western blot for NLRP3. Lane 1: HCEC cell lysate under 310mOsm; Lane2: HCEC cell lysate under 450mOsm; Lane2: HCEC cell lysate under 450mOsm with hADSC-Evs.

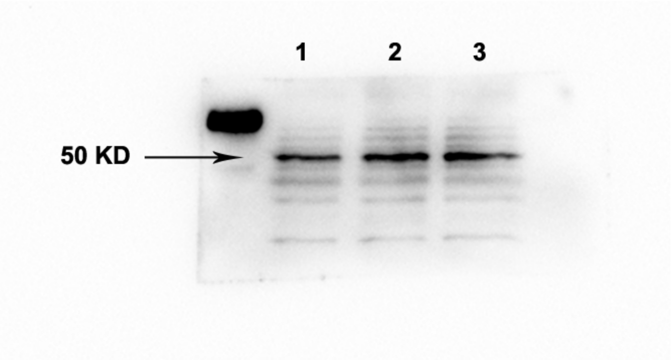

Figure S7: Full length gel of western blot for pro-caspase-1(pro-casp-1). Lane 1: HCEC cell lysate under 310mOsm; Lane2: HCEC cell lysate under 450mOsm; Lane2: HCEC cell lysate under 450mOsm with hADSC-Evs.

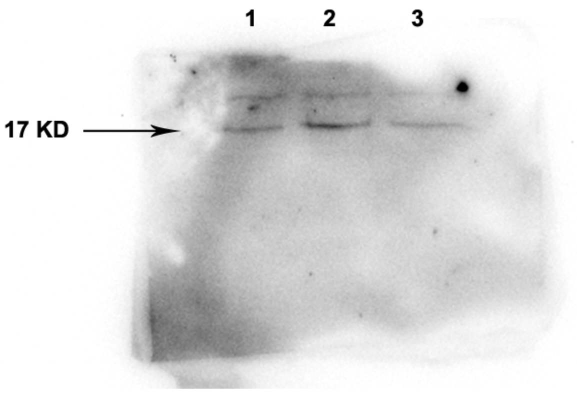

Figure S8: Full length gel of western blot for cleaved-caspase-1(cleaved casp-1). Lane 1: HCEC cell lysate under 310mOsm; Lane2: HCEC cell lysate under 450mOsm; Lane2: HCEC cell lysate under 450mOsm with hADSC-Evs.

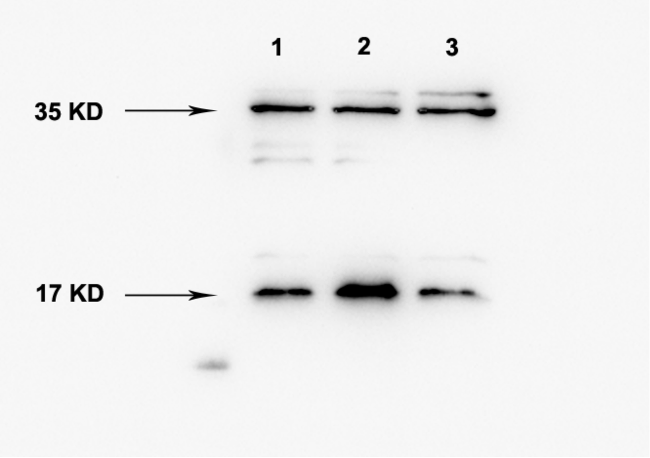

Figure S9: Full length gel of western blot for pro-IL-1βand cleavedIL-1β. Lane 1:

HCEC cell lysate under 310mOsm; Lane2: HCEC cell lysate under 450mOsm; Lane2: HCEC cell lysate under 450mOsm with hADSC-Evs.

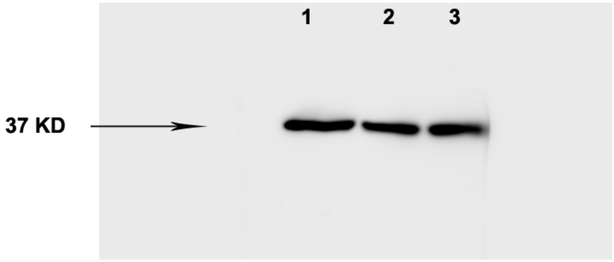

Figure S10: Full length gel of western blot for GAPDH. Lane 1: HCEC cell lysate under 310mOsm; Lane2: HCEC cell lysate under 450mOsm; Lane2: HCEC cell lysate under 450mOsm with hADSC-Evs.
